# Supplementary material for: A Balanced Time Perspective and Burnout Syndrome in the Corporate World
Source: Int J Environ Res Public Health. 2022 Nov 4;19(21):14466. doi: 10.3390/ijerph192114466 (PMC9657483; doi:10.3390/ijerph192114466)
Supplement: Supplementary file 1 [file ijerph-19-14466-s001.zip › ijerph-1919129-supplementary.pdf]

Supplement S1: Zimbardo Time Perspective Inventory

The following survey consists of 15 statements intended at determining your individual time perspective. Please carefully read each statement and honestly answer to what extent the statement describes you:

1. Strongly disagree,
2. Disagree,
3. Neither agree nor disagree,
4. Agree,
5. Strongly agree

1. I believe that getting together with one's friends to party is one of life's important pleasures.
2. Fate determines much in my life.
3. It gives me pleasure to think about my past.
4. Meeting tomorrow's deadline and doing other necessary work comes before tonight's play.
5. Since whatever will be will be, it doesn't really matter what I do.
6. I enjoy stories about how things used to be in the "good old times".
7. Painful past experiences keep being replayed in my mind.
8. Happy memories of good times spring readily to mind.
9. It is important to put excitement in my life.
10. I've made mistakes in the past that I wish that I could undo.
11. It's hard for me to forget unpleasant images of my youth.
12. It doesn't make sense to worry about the future, since there is nothing that I can do about it anyway.
13. I complete projects on time by making steady progress.
14. I am able to resist temptations when I know that there is work to be done.
15. I prefer friends who are spontaneous rather than predictable.

Supplement S2: Maslach Burnout Inventory

The following 22 questions are intended to determine your individual attitude towards work. In this part, to answer the question, choose the frequency of the feelings in question.

0- never

1- a few times a year or less

2- once every few months

3- once a month

4- a few times a month

5- a few times a week

6- everyday

1. I feel emotionally exhausted from my work
2. I feel worn out by the end of the work day
3. When I wake up, I feel tired just thinking about the work day
4. Everyday work with others is exhausting for me
5. I feel burned out by my work
6. I feel frustrated by my work
7. I think that I work too hard
8. Being in direct contact with others is too stressful for me
9. I feel that I reached the limit of knowledge in my field
10. I feel that I treat some co-workers as if they were objects
11. I've gotten indifferent towards my co-workers
12. I fear that my work is making me less sensitive
13. I do not care too much about what happens to some of my co-workers
14. I have a feeling that some people at work think that I'm at fault for their failures
15. I find it easy to assume what my co-workers think about various subjects
16. I manage to effectively solve the problems of my colleagues / customers
17. I feel that I am making a positive impact on my life, through my work
18. I feel that I have a lot of energy within me
19. I can easily influence an easygoing atmosphere at work
20. I feel alive when I work with colleagues / customers
21. I have reached many substantial goals at work
22. I stay calm during emotional difficulties at work
